# Intestinal Growth in Glucagon Receptor Knockout Mice Is Not Associated With the Formation of AOM/DSS-Induced Tumors

**DOI:** 10.3389/fendo.2021.695145

**Published:** 2021-05-24

**Authors:** Jenna Elizabeth Hunt, Mohammad Yassin, Jørgen Olsen, Bolette Hartmann, Jens Juul Holst, Hannelouise Kissow

**Affiliations:** ^1^ Department of Biomedical Sciences, Faculty of Health and Medical Sciences, University of Copenhagen, Copenhagen, Denmark; ^2^ Department of Cellular and Molecular Medicine, Faculty of Health and Medical Sciences, University of Copenhagen, Copenhagen, Denmark; ^3^ Department of Biomedical Sciences and Novo Nordisk Foundation Center for Basic Metabolic Research, Faculty of Health and Medical Sciences, University of Copenhagen, Copenhagen, Denmark

**Keywords:** GLP-2, AOM/DSS, glucagon receptor knockout, mice, intestinal growth

## Abstract

Treatment with exogenous GLP-2 has been shown to accelerate the growth of intestinal adenomas and adenocarcinomas in experimental models of colonic neoplasia, however, the role of endogenous GLP-2 in tumor promotion is less well known. Mice with a global deletion of the glucagon receptor (Gcgr^-/-^) display an increase in circulating GLP-1 and GLP-2. Due to the intestinotrophic nature of GLP-2, we hypothesized that Gcgr^-/-^ mice would be more susceptible to colonic dysplasia in a model of inflammation-induced colonic carcinogenesis. Female Gcgr^-/-^ mice were first characterized for GLP-2 secretion and in a subsequent study they were given a single injection with the carcinogen azoxymethane (7.5 mg/kg) and treated with dextran sodium sulfate (DSS) (3%) for six days (n=19 and 9). A cohort of animals (n=4) received a colonoscopy 12 days following DSS treatment and all animals were sacrificed after six weeks. Disruption of glucagon receptor signaling led to increased GLP-2 secretion (p<0.0001) and an increased concentration of GLP-2 in the pancreas of Gcgr^-/-^ mice, coinciding with an increase in small intestinal (p<0.0001) and colonic (p<0.05) weight. Increased villus height was recorded in the duodenum (p<0.001) and crypt depth was increased in the duodenum and jejunum (p<0.05 and p<0.05). Disruption of glucagon receptor signaling did not affect body weight during AOM/DSS treatment, neither did it affect the inflammatory score assessed during colonoscopy or the number of large and small adenomas present at the end of the study period. In conclusion, despite the increased endogenous GLP-2 secretion Gcgr^-/-^ mice were not more susceptible to AOM/DSS-induced tumors.

## Introduction

The glucagon-like peptides (GLPs) are post-translational cleavage products of the precursor polypeptide proglucagon (1, 2). Proglucagon is processed in a tissue-specific manner, giving rise to glicentin/oxyntomodulin, GLP-1 and GLP-2 in the intestine and brain, and glucagon and the major proglucagon fragment in the pancreas (2, 3). GLP-2 exerts its effect primarily in the intestine where it controls mucosal growth and adaptation (1, 4, 5). Administration of exogenous GLP-2 increases intestinal proliferation, decreases apoptosis (6), improves nutrient absorption (7), enhances barrier function (8) and intestinal blood flow (9, 10), and it can be demonstrated that endogenous GLP-2 mediates adaptive regrowth after a period of nutrient deprivation (11). These intestinotrophic features of GLP-2 have guided the hormone’s therapeutic potential for the treatment of intestinal injury, culminating in the FDA approval of the DPP-4 resistant human GLP-2 analog teduglutide for the treatment of adults and children aged 1 and over for short bowel syndrome, who are receiving parenteral support (12).

Due to the observed tropic effects of exogenous GLP-2, the potential for growth acceleration and malignant transformation of subclinical neoplasia remains a question for the safety of treatment with GLP-2 receptor agonists. Several studies have examined the effects of GLP-2 on the development of neoplastic changes in the intestines of mice (13–15) using multiple modes of tumorigenesis including pro-carcinogens azoxymethane (AOM), 1,2-dimethylhydrazine (DMH) as well as Apc^Min/+^ mice. Yet, the lack of consensus in the literature regarding the tumor-promoting effect of GLP-2 warrants further investigations; in addition, the effects of chronically elevated levels of endogenous GLP-2 and its potential contribution to neoplastic development have never been investigated.

Partial or complete blockage of glucagon action, achieved through genetic loss (16–18) or pharmacological blockage of glucagon signaling (17, 19) is associated with pancreatic GLP-1 production and increased circulating GLP-1 in mice, most likely due to pancreatic α-cell hyperplasia (20). Consequently, these mice display decreased blood glucose (21), improved glucose tolerance, enhanced glucose-stimulated insulin secretion (17), and resistance to streptozotocin-induced diabetes (22). The consequences of attenuating glucagon receptor signaling on GLP-2 and the assessment of impact is less well known, but it has been demonstrated that a small fraction of the major proglucagon fragment is normally processed to GLP-2 (23).

In the present study, we assessed the use of Gcgr^–/–^ mice as a model of chronically elevated GLP-2 secretion. We characterized the tissue-specific production of GLP-2 in the pancreas, intestine and plasma and hypothesized that these changes would correlate with a characteristic GLP-2-induced growth response. Finally, we used AOM/DSS treated Gcgr^–/–^ mice to assess the development of colonic tumorigenesis in the presence of increased endogenous GLP-2.

## Materials and Methods

### Animals

All experiments were conducted following the guidelines of Danish legislation governing animal experimentation (1987) and with permission from the Danish Animal Experiments Inspectorate (license no. 2013-15-2934-00833). Glucagon receptor knockout (Gcgr^−/−^) mice C57BL/^6Gcgrtm1Mjch^ were bred in-house with permission from Dr. Maureen Charron as detailed previously (16). Animals used in experimentation were between 8-10 weeks old and Gcgr^+/+^ littermates were used as controls. All mice were housed in individually ventilated cages in a standard 12:12 h light-dark cycle with free access to water and standard chow. Mice were housed in groups of 6-8 independent of genotype.

### Intestinal Characterization of *Gcgr^−/−^* Mice

Unfasted female *Gcgr^−^* ^/−^ mice (n=8) and their *Gcgr^+/+^* littermates (n=10) were anesthetized with an intraperitoneal injection of ketamine (90 mg/kg) (MSD Animal Health, Madison, New Jersey, USA) and xylazine (10 mg/kg) (Rompun Vet, Bayer Animal Health, Leverkusen, Germany), body weight was recorded and blood was drawn from vena cava and transferred to EDTA coated tubes containing a dipeptidyl peptidase-4 inhibitor (0.01mmol/L valine pyrrolidide (ValPyr), final concentration) (Novo Nordisk, Denmark). Plasma samples were centrifuged at 1200 g for 15 min at 4°C then stored at -20°C until processing. The small and large intestine was removed, flushed with saline and weighed (24). Tissue from duodenum, jejunum, ileum and mid colon was fixed in 10% neutral formalin buffer (Cell Path Ltd, Powys, United Kingdom) for 24 h and afterward transferred to 70% alcohol until further processing for morphometry. Tissue from duodenum, jejunum, ileum, mid colon and pancreas was snap-frozen and stored at -80°C for gut hormone analysis.

In a separate study, unfasted female and male *Gcgr^−^* ^/−^ mice (n=16) and their *Gcgr^+/+^* littermates (n=15) were anesthetized as above and plasma was collected as described above.

### Histology

Formalin-fixed tissue was dehydrated and paraffin-embedded. Histological transverse sections of 4 μm were cut and stained with hematoxylin/eosin. Villus height and crypt depth was measured in at least 20 well-oriented villi and crypts per animal (24). Slides were examined with a light microscope connected to a camera (Zeiss Axio Lab.A1, Brock & Michelsen, Birkeroed, Denmark). Morphology was analyzed using Zeiss Zen lite software (Carl Zeiss Microscopy GmbH, Göttingen, Germany). All measurements and evaluations were performed with the observer blinded from the origin of the tissue.

### Pancreatic and Intestinal Protein Extraction, Plasma Extraction and Measurement of GLP-2

Snap-frozen tissue was subject to peptide extraction carried out as previously described (25). In brief, the protein was extracted by homogenization in 1% trifluoroacetic acid (TFA) (Thermofisher Scientific, Massachusetts, USA) and the concentration of protein was determined using the Pierce BCA Protein Assay Kit (Thermofisher Scientific). The peptide was purified by solid phase extraction using tc18 cartridges (Waters, Massachusetts, USA) which were eluted using 70% ethanol containing 0.1% TFA. The eluates were air-dried overnight using a blow system and reconstituted in 1 ml of assay buffer (phosphate buffer 80 mM, 0.1% human serum albumin, EDTA 10 mM, pH 7.5) containing 0.01 mM ValPyr. Intact, biologically active GLP-2 was quantified by radioimmunoassay utilizing antiserum #92160 specific for an intact N-terminus of GLP-2 (1–33) (2). Plasma measurements were made from two pooled plasma samples and were extracted with 75% ethanol before analysis.

### Establishment of the AOM/DSS Model

Female *Gcgr^−^* ^/−^ mice (n=19) and their *Gcgr^+/+^* littermates (n=8) received an intraperitoneal injection of azoxymethane (7.4 mg/kg) (Sigma-Aldrich, Denmark), followed by one cycle of 3% dextran sulfate sodium (DSS) (M_r_ ~40,000) (Sigma-Aldrich, Denmark) for six days commencing day eight. Mice were weighed on day 1, 4, 8, 10, 11, 12, 13, 16, 18, 27, 30, 38 and 44. All mice were euthanized on day 44.

### Endoscopic Investigation

Twelve days after DSS treatment *Gcgr^−^* ^/−^ mice (n=4) and their *Gcgr^+/+^* littermates (n=4) were anesthetized with isoflurane (Baxter, Lillerod, Denmark; flow concentration: (1.5% vol./vol.) and subjected to high-resolution colonoscopy using the COLOVIEW mini-endoscopic system (Karl Storz, Tuttlingen, Germany) according to the Becker et al., 2006 protocol (26). The observer was blinded as to the genotype of the mouse, and colons were assessed using the murine endoscopic index of colitis severity (MEICS), with a maximal score of 15.

### Macroscopic Analysis of Early Colon Adenomas

After euthanasia by cervical dislocation, the colons were flushed through the rectum with PBS, followed by ice-cold 4% paraformaldehyde. Colons were fixed for 3-5 min with paraformaldehyde. The colons were then removed, cut longitudinally and pinned to a polyethylene plate. The samples were further fixated for 24 h in 4% paraformaldehyde and then transferred to 70% ethanol until further analysis. The colons were washed in distilled water and stained with 0.2% methylene blue for 30 min and examined using a stereomicroscope. The number of adenomas ≤ 2 mm in diameter and those larger were counted. The investigator was blinded concerning the origin of the tissue.

### Statistical Analysis

All statistics were performed using GraphPad Prism 8. Statistical evaluation of the data was carried out using two-sided, unpaired t tests when comparing two independent groups. Survival curves were drawn using the Kaplan-Meier method and analyzed using the log-rank test. Values of p<0.05 were considered significant and all data in the text and graphs are presented as mean ± SEM.

## Results

### Biochemical Characterization of *Gcgr^−/−^* and *Gcgr^+/+^* Mice

**Figure 1:**
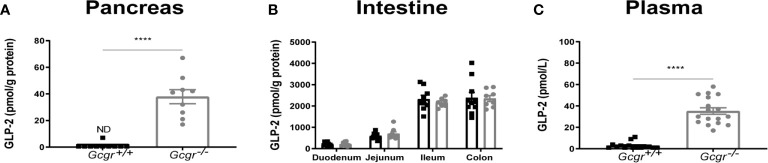
Biochemical characterization of *Gcgr^−/−^* and *Gcgr^+/+^* mice. Extracted GLP-2 (1-33) in **(A)** the pancreas and **(B)** intestine normalized to grams (g) protein determined by BCA assay. **(C)** Plasma levels of GLP-2 (1-33). *Gcgr^+/+^* mice are shown in black and *Gcgr^−/−^* mice are shown in grey. Data were compared using a two-sided, unpaired students t-test and presented as means ± SEM. ****p value <0.0001, ND, non-detectable.

Extractable GLP-2 in the pancreas could not be detected in *Gcgr^+/+^* mice but was present in the *Gcgr^−/−^* mice at a concentration of 38 ± 15 pmol/g protein (p<0.0001) (**Figure 1A**). Extractable intestinal GLP-2 was not affected by genotype (**Figure 1B**). Secreted GLP-2 measured in the plasma was found to be 35 ± 13 pmol/L in the *Gcgr^−^* ^/−^ mice. This was highly significant compared to 3 ± 1 pmol/L in the *Gcgr^+/+^* mice (p<0.0001) (**Figure 1C**).

### Body Weight and Morphometric Characterization of the Intestine of *Gcgr^−/−^* and *Gcgr^+/+^* Mice

**Figure 2:**
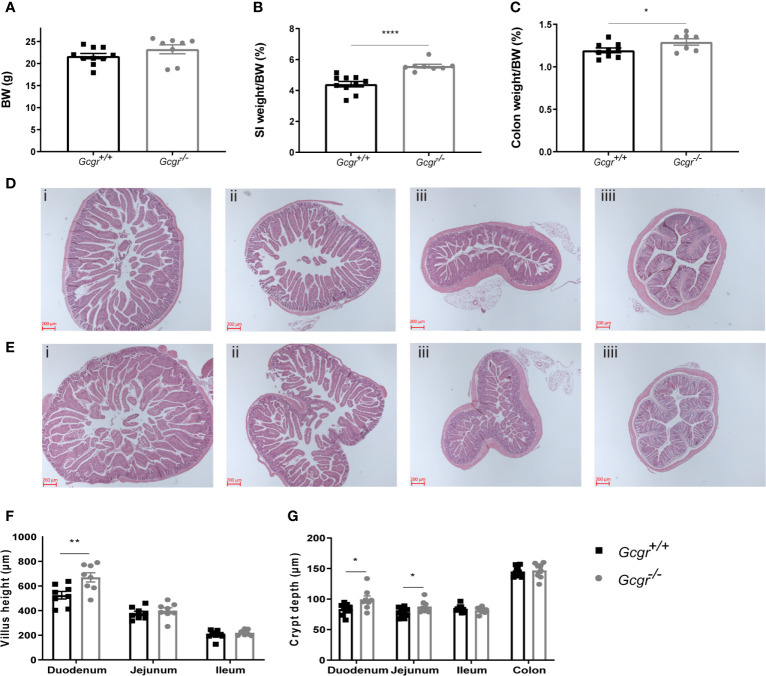
Morphometric characterization of the intestine of *Gcgr^−/−^* and *Gcgr^+/+^* mice. **(A)** Body weight (BW). **(B)** Small intestinal (SI) weight and **(C)** colon weight normalized to BW. **(D)** Hematoxylin and eosin-stained intestinal tissue, from *Gcgr^+/+^* mice **(Di)** duodenum, **(Dii)** jejunum, **(Diii)** ileum and **(Diiii)** colon; and **(E)** *Gcgr^-/-^* mice **(Ei)** duodenum, **(Eii)** jejunum, **(Eiii)** ileum and **(Eiiii)** colon. **(F)** Villus height and **(G)** crypt depth estimations. Data were compared using a two-sided, unpaired students t-test and presented as means ± SEM. *p value <0.05, **p value <0.01, ****p value <0.0001.

The body weight of *Gcgr^−^* ^/−^ mice did not differ from their *Gcgr^+/+^* littermates (**Figure 2A**). The SI and colon weight, normalized to body weight, was 25% and 8% larger in *Gcgr^−/−^* mice compared to *Gcgr^+/+^*, (p<0.0001 and p<0.05) (**Figures 2B, C**). Villus height was significantly higher in the duodenum of *Gcgr^−^* ^/−^ mice compared to *Gcgr^+/+^* (p<0.01) but did not differ in the jejunum or ileum (**Figure 2F**) as visualized in the histological photographs (**Figures 2D, E**). Crypt depth was significantly deeper in the duodenum and jejunum of *Gcgr^−^* ^/−^ mice compared to *Gcgr^+/+^* (p<0.05 and p<0.05) (**Figure 2G**) but the crypts in the ileum and colon did not differ (**Figure 2G**).

### AOM/DSS Model in Gcgr^−/−^ Mice

**Figure 3:**
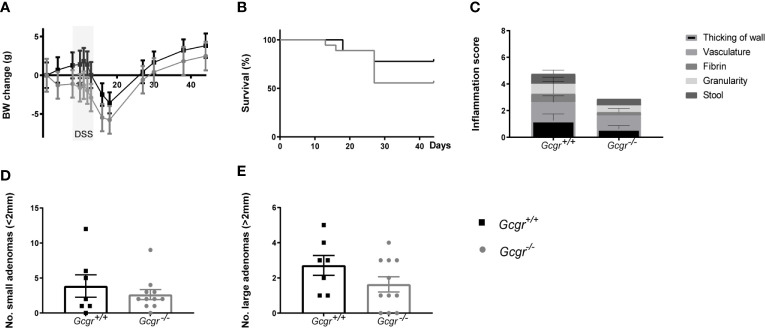
*AOM/DSS model in Gcgr^−^* ^/−^ and Gcgr^+/+^ mice. Female *Gcgr^−^* ^/−^ mice (n=19) and their *Gcgr^+/+^* littermates (n=8) received an intraperitoneal injection of azoxymethane (7.4 mg/kg), followed by one cycle of 3% DSS commencing day eight for six days. Body weight (BW) **(A)** was recorded once or twice a week. **(B)** Survival probability during the period of experimentation. **(C)** Murine endoscopic index of colitis severity, with a maximal score of 15. **(D)** The number of small adenomas per colon defined as smaller than 2 mm in diameter. **(E)** The number of large adenomas per colon defined as larger than 2 mm in diameter. Data were compared using a two-sided, unpaired students t-test or log-rank test and presented means ± SEM.

Throughout the experiment, the body weight did not differ greatly between *Gcgr^-/-^* and *Gcgr^+/+^* mice, but the body weight characteristically fell and recovered following DSS treatment regardless of genotype (**Figure 3A**). The final body weight did not differ between *Gcgr^−^* ^/−^ and *Gcgr^+/+^* mice. Genotype did not affect the survival probability following AOM/DSS (**Figure 3B**). Colonic mucosal damage, scored by colonoscopy 12 days after DSS treatment, did not differ between *Gcgr^-/-^* and *Gcgr^+/+^* mice (**Figure 3C**). After 6 weeks, all animals had the presence of adenomas, predominantly located in the distal part of the colon. The number of both small, defined as ≤ 2 mm in diameter, and large adenomas, defined as > 2 mm in diameter was the same in *Gcgr^-/-^* and *Gcgr^+/+^* mice (**Figures 3D, E**).

## Discussion

Teduglutide, a stabilized version of GLP-2, has been approved for the treatment of adult and pediatric (1 year and older) short bowel syndrome in patients requiring parenteral support (12). Benefits to patients include an increase in intestinal wet weight and absorption (27) leading to a reduction in parenteral nutrition volume requirements, but exogenous GLP-2 administration in animal models has been linked to the acceleration of neoplastic growth (13, 14). This highlights the urgency of further research into the role of GLP-2 and malignancy. Here, we used the *Gcgr^-/-^* mouse as a model for elevated GLP-2 and assessed colonic dysplasia using the AOM/DSS model of inflammation-induced colonic carcinogenesis.

We investigated the intestinal parameters in the *Gcgr^-/-^* animals. The weight of the small intestine was increased by 25% in the *Gcgr^-/-^* mice. Morphological examination revealed increased villus height and crypt depth in the proximal portion. Also, the weight of the colons of the *Gcgr^-/-^* mice were found to be larger than normal. Such a growth response mirrors that seen following GLP-2 treatment in animal models (26–28). Next, we investigated the plasma concentration of GLP-2 and found that this was highly elevated in the *Gcgr^-/-^* mice. Proglucagon is produced in both the pancreas and in the intestines, therefore we investigated the tissue concentration in these tissues. Even though we did find that the intestines were hypertrophic, GLP-2 measurements from the intestine showed the typical distribution with the highest concentrations measured in the colon followed by the ileum with no differences between knockout and wild type animals. However, we did find that the pancreas had an elevated concentration of GLP-2. We have previously described that *Gcgr^-/-^* mice showed alpha-cell hyperplasia and hypertrophy, and increased pancreatic concentrations of GLP-1 and glucagon (20). In the normal state, proglucagon is primarily processed to glucagon by prohormone convertase (PC1/3) in the alpha cells (3) and GLP-1 and GLP-2 are mainly produced in the intestinal L cells by PC2 cleavage, but small amounts of fully processed GLP-2 are found in the pancreas (23). As previously observed (20), GLP-1 concentrations were rather low compared to the very increased glucagon levels (<1%), and the increase in both GLP-1 and GLP-2 is assumed to be a consequence of extreme proglucagon expression in the knockout mice. It would seem fair to assume that the massive intestinal growth would be a consequence of the increased level of GLP-2, however, we cannot from our data exclude any other reasons.

Taken together we proposed the *Gcgr^-/-^* mouse as a model of increased endogenous GLP-2, which can be used to investigate chronically elevated levels of endogenous GLP-2 and its potential contribution to neoplastic development. Several methodologies exist to induce colon carcinogenesis, yet the AOM/DSS model closely resembles the pathogenesis observed in human colorectal cancer; characterized by frequent tumors located in the distal colon and beginning with polypoidal growth (29). In this study, we show that during the acute inflammation phase following the chemically-induced mucosal injury (12 days following DSS treatment) all mice displayed features of neoplastic growth but the loss of glucagon signalling did not affect the MEICS-score. Additionally, six weeks post AOM injection the number of adenomas was unaffected by the missing glucagon receptor expression. These observations contrast the outcomes of other carcinogen-induced tumorigenesis studies that describe an increase in dysplastic changes following teduglutide treatment (13, 14) and instead support conclusions drawn from experimentation utilizing native GLP-2, wherein there were no increases in malignancy following GLP-2 treatment, utilizing AOM, DMH and the genetic model of human intestinal cancer Apc^Min/+^ mice (15). However, the functionality of this mouse strain stems from germline mutations in the *Gcgr* gene which potentially could complicate the interpretation of our data by unanticipated compensatory adaptations arising in mice with germline gene deletions. To combat this issue, we suggest further exploration utilizing long-term glucagon antagonist and assessment of growth and gut hormone content.

To conclude, our results show that although elevated levels of native GLP-2 were associated with a massive growth of both the small intestine and colon, it did not contribute to aggravation of neoplastic growth.

## Data Availability Statement

The original contributions presented in the study are included in the article/supplementary material. Further inquiries can be directed to the corresponding author.

## Ethics Statement

The animal studies were reviewed and approved by the Danish Animal Experiments Inspectorate.

## Author Contributions

JEH and HK planned and designed the study. MY and JO provided technical support to the study. JEH, MY, BH and HK performed the experiments. JEH analyzed the results and JEH, JJH and HK interpreted the results of the experiment. JEH drafted the manuscript. All authors contributed to the article and approved the submitted version.

## Funding

This work was supported by the Lundbeck Foundation (Grant No. R263-2017-3740), the Novo Nordisk Foundation Center for Basic Metabolic Research (Novo Nordisk Foundation, Denmark), Agnes and Poul Friis Fondation and Læge Sofus Carl Emil Friis og Hustru Olga Doris Friis’ Legat. The funding sources were not involved in the study design, collection, analysis or interpretation of data.

## Conflict of Interest

MY and JO are the founders of Enterotarget Aps. JJH and BH are the co-founders of Bainan Biotech.

The remaining authors declare that the research was conducted in the absence of any commercial or financial relationships that could be construed as a potential conflict of interest.

The reviewer GM declared a past co-authorship with the authors BH, JJH to the handling editor.

## References

[1] Drucker DJ Ehrlich P Asat SL Brubaker PL. Induction of Intestinal Epithelial Proliferation by Glucagon-Like Peptide 2. Proc Natl Acad Sci USA (1996) 93:7911–6. 10.1073/pnas.93.15.7911 PMC38848 8755576

[2] Hartmann B Johnsen AH Ørskov C Adelhorst K Thim L Holst JJ. Structure, Measurement, and Secretion of Human Glucagon-Like Peptide-2. Peptides (2000) 21:73–80. 10.1016/S0196-9781(99)00176-X 10704722

[3] Rouille Y Westermark G Martin SK Steiner DF. Proglucagon is Processed to Glucagon by Prohormone Convertase PC2 in Alpha TC1-6 Cells. Proc Natl Acad Sci (1994) 91:3242–6. 10.1073/pnas.91.8.3242 PMC43552 8159732

[4] Jeppesen PB Hartmann B Thulesen J Graff J Lohmann J Hansen BS. Glucagon-Like Peptide 2 Improves Nutrient Absorption and Nutritional Status in Short-Bowel Patients With No Colon. Gastroenterology (2001) 120:806–15. 10.1053/gast.2001.22555 11231933

[5] Thulesen J Hartmann B Kissow H Jeppesen P Orskov C Holst J. Intestinal Growth Adaptation and Glucagon-Like Peptide 2 in Rats With Ileal–Jejunal Transposition or Small Bowel Resection. Dig Dis Sci (2001) 46:379–88. 10.1023/a:1005572832571 11281189

[6] Burrin DG Stoll B Guan X Cui L Chang X Holst JJ. Glucagon-Like Peptide 2 Dose-Dependently Activates Intestinal Cell Survival and Proliferation in Neonatal Piglets. Endocrinology (2005) 146:22–32. 10.1210/en.2004-1119 15486229

[7] Brubaker P Izzo A Hill M Drucker DJ. Intestinal Function in Mice With Small Bowel Growth Induced by Glucagon-Like Peptide-2. Am J Physiol Metab (1997) 272:E1050–8. 10.1152/ajpendo.1997.272.6.E1050 9227451

[8] Kouris GJ Liu Q Rossi H Djuricin G Gattuso P Nathan C. The Effect of Glucagon-Like Peptide 2 on Intestinal Permeability and Bacterial Translocation in Acute Necrotizing Pancreatitis. Am J Surg (2001) 181:571–5. 10.1016/s0002-9610(01)00635-3 11513789

[9] Guan X Karpen HE Stephens J Bukowski JT Niu S Zhang G. Glp-2 Receptor Localizes to Enteric Neurons and Endocrine Cells Expressing Vasoactive Peptides and Mediates Increased Blood Flow. Gastroenterology (2006) 130:150–64. 10.1053/j.gastro.2005.11.005 16401478

[10] Stephens J Stoll B Cottrell J Chang X Helmrath M Burrin DG. Glucagon-Like Peptide-2 Acutely Increases Proximal Small Intestinal Blood Flow in TPN-Fed Neonatal Piglets. Am J Physiol Regul Integr Comp Physiol (2005) 290:283–9. 10.1152/ajpregu.00588.2005 16166200

[11] Shin ED Estall JL Izzo A Drucker DJ Brubaker PL. Mucosal Adaptation to Enteral Nutrients is Dependent on the Physiologic Actions of Glucagon-Like Peptide-2 in Mice. Gastroenterology (2005) 128:1340–53. 10.1053/j.gastro.2005.02.033 15887116

[12] Takeda Pharmaceutical Company Limited. *U.S. FDA Approves GATTEX® (teduglutide) for Children 1 Year of Age and Older With Short Bowel Syndrome*. (2019). Available at: https://www.takeda.com/en-us/newsroom/news-releases/2019/u.s.-fda-approves-gattex-teduglutide-for-children-1-year-of-age-and-older-with-short-bowel-syndrome-sbs/ (Accessed May 12, 2021).

[13] Iakoubov R Lauffer LM Trivedi S Kim YIJ Brubaker PL. Carcinogenic Effects of Exogenous and Endogenous Glucagon-Like Peptide-2 in Azoxymethane-Treated Mice. Endocrinology (2009) 150:4033–43. 10.1210/en.2009-0295 19497974

[14] Thulesen J Hartmann B Hare KJ Kissow H Ørskov C Holst JJ. Glucagon-Like Peptide 2 (GLP-2) Accelerates the Growth of Colonic Neoplasms in Mice. Gut (2004) 53:1145–50. 10.1136/gut.2003.035212 PMC1774162 15247183

[15] Koehler JA Harper W Barnard M Yusta B Drucker DJ. Glucagon-Like Peptide-2 Does Not Modify the Growth or Survival of Murine or Human Intestinal Tumor Cells. Cancer Res (2008) 68:7897–904. 10.1158/0008-5472.CAN-08-0029 PMC3606135 18829546

[16] Gelling RW Du XQ Dichmann DS Rømer J Huang H Cui L. Lower Blood Glucose, Hyperglucagonemia, and Pancreatic α Cell Hyperplasia in Glucagon Receptor Knockout Mice. Proc Natl Acad Sci USA (2003) 100:1438–43. 10.1073/pnas.0237106100 PMC298791 12552113

[17] Gu W Winters KA Motani AS Komorowski R Zhang Y Liu Q. Glucagon Receptor Antagonist-Mediated Improvements in Glycemic Control are Dependent on Functional Pancreatic GLP-1 Receptor. AJP Endocrinol Metab (2010) 299:E624–32. 10.1152/ajpendo.00102.2010 20647556

[18] Gu W Yan H Winters KA Komorowski R Vonderfecht S Atangan L. Long-Term Inhibition of the Glucagon Receptor With a Monoclonal Antibody in Mice Causes Sustained Improvement in Glycemic Control, With Reversible α-Cell Hyperplasia and Hyperglucagonemia. J Pharmacol Exp Ther (2009) 331:871–81. 10.1124/jpet.109.157685 19720878

[19] Ali S Charron MJ Drucker DJ Ali S Lamont BJ Charron MJ. Dual Elimination of the Glucagon and GLP-1 Receptors in Mice Reveals Plasticity in the Incretin Axis. J Clin Invest (2011) 121:1917–29. 10.1172/JCI43615 PMC3083792 21540554

[20] Svendsen B Larsen O Gabe MBN Christiansen CB Rosenkilde MM Drucker DJ. Insulin Secretion Depends on Intra-Islet Glucagon Signaling. Cell Rep (2018) 25:1127–1134.e2. 10.1016/j.celrep.2018.10.018 30380405

[21] Galsgaard KD Winther-Sørensen M Ørskov C Kissow H Poulsen SS Vilstrup H. Disruption of Glucagon Receptor Signaling Causes Hyperaminoacidemia Exposing a Possible Liver - Alpha-Cell Axis. Am J Physiol - Endocrinol Metab (2017) 314:E93–103. 10.1152/ajpendo.00198.2017 28978545 PMC6048389

[22] Conarello SL Jiang G Mu J Li Z Woods J Zycband E. Glucagon Receptor Knockout Mice Are Resistant to Diet-Induced Obesity and Streptozotocin-Mediated Beta Cell Loss and Hyperglycaemia. Diabetologia (2007) 50:142–50. 10.1007/s00125-006-0481-3 17131145

[23] Holst JJ Bersanis M Johnsenn AH Kofodll H Hartmanns B. Proglucagon Processing in Porcine and Human Pancreas. J Biol Chem (1994) 269:18827–33. 10.1016/S0021-9258(17)32241-X 8034635

[24] Billeschou A Hunt J Kissow H. Important Endpoints and Proliferative Markers to Assess Small Intestinal Injury and Adaptation Using a Mouse Model of Chemotherapy-Induced Mucositis. J Vis Exp (2019) 147:e59236. 10.3791/59236 31132057

[25] Albrechtsen NJW Kuhre RE Toräng S Holst JJ. The Intestinal Distribution Pattern of Appetite and Glucose Regulatory Peptides in Mice, Rats and Pigs. BMC Res Notes (2016) 9:1–7. 10.1186/s13104-016-1872-2 26830025 PMC4736122

[26] Becker C Fantini MC Neurath MF. High Resolution Colonoscopy in Live Mice. Nat Protoc (2007) 1:2900–4. 10.1038/nprot.2006.446 17406549

[27] Jeppesen PB Sanguinetti EL Buchman A Howard L Scolapio JS Ziegler TR. Teduglutide (ALX-0600), a Dipeptidyl Peptidase IV Resistant Glucagon-Like Peptide 2 Analogue, Improves Intestinal Function in Short Bowel Syndrome Patients. Gut (2005) 54:1224–31. 10.1136/gut.2004.061440 PMC1774653 16099790

[28] Taylor-Edwards C Burrin DG Holst JJ Mcleod KR HD L. Glucagon-Like Peptide-2 (GLP-2) Increases Small Intestinal Blood Flow and Mucosal Growth in Ruminating Calves. J Dairy Sci (2011) 94:888–9. 10.3168/jds.2010-3540 21257057

[29] Takuji T. Colorectal Carcinogenesis: Review of Human and Experimental Animal Studies. J Carcinog (2009) 8:5. 10.4103/1477-3163.49014 19332896 PMC2678864

